# Within-Host Fitness and Antigenicity Shift Are Key Factors Influencing the Prevalence of Within-Host Variations in the SARS-CoV-2 *S* Gene

**DOI:** 10.3390/v17030362

**Published:** 2025-03-02

**Authors:** Binbin Xi, Zhihao Hua, Dawei Jiang, Zixi Chen, Jinfen Wei, Yuhuan Meng, Hongli Du

**Affiliations:** 1 School of Biology and Biological Engineering, South China University of Technology, Guangzhou 510006, China; 2 Guangzhou KingMed Transformative Medicine Institute, KingMed School of Laboratory Medicine, Guangzhou Medical University, Guangzhou 510220, China

**Keywords:** SARS-CoV-2, iSNVs, prevalence, within-host fitness, antigenicity shift

## Abstract

Within-host evolution plays a critical role in shaping the diversity of SARS-CoV-2. However, understanding the primary factors contributing to the prevalence of intra-host single nucleotide variants (iSNVs) in the viral population remains elusive. Here, we conducted a comprehensive analysis of over 556,000 SARS-CoV-2 sequencing data and prevalence data of different SARS-CoV-2 S protein amino acid mutations to elucidate key factors influencing the prevalence of iSNVs in the SARS-CoV-2 *S* gene. Within-host diversity analysis revealed the presence of mutational hotspots within the *S* gene, mainly located in NTD, RBD, TM, and CT domains. Additionally, we generated a single amino acid resolution selection status map of the S protein. We observed a significant variance in within-host fitness among iSNVs in the S protein. The majority of iSNVs exhibited low to no within-host fitness and displayed low alternate allele frequency (AAF), suggesting that they will be eliminated due to the narrow transmission bottleneck of SARS-CoV-2. Notably, iSNVs with moderate AAFs (0.06–0.12) were found to be more prevalent than those with high AAFs. Furthermore, iSNVs with the potential to alter antigenicity were more prevalent. These findings underscore the significance of within-host fitness and antigenicity shift as two key factors influencing the prevalence of iSNVs in the SARS-CoV-2 *S* gene.

## 1. Introduction

The continuous evolution of SARS-CoV-2 gives rise to new lineages that harbor mutations capable of increasing viral fitness [1]. These mutations, particularly those enhancing immune escape capabilities, pose great threats to human health [2,3]. Therefore, it is crucial to understand the principles underlying the emergence of prevalent mutations within the SARS-CoV-2 population.

The emergence of prevalent mutations can be delineated into four processes: the de novo generation of intra-host single nucleotide variants (iSNVs), within-host evolution, transmission, and population-level competition. Following the transmission of a homogenous SARS-CoV-2 population into a new host, iSNVs are generated de novo within the viral genome, resulting in a genetically heterogeneous viral population known as a quasispecies [4]. The process of the de novo iSNV generation of SARS-CoV-2 is primarily dominated by ROS and host-directed RNA-editing proteins, specifically the activation-induced cytidine deaminase/apolipoprotein B mRNA-editing enzyme catalytic polypeptide-like (AID/APOBEC) protein family and the adenosine deaminase acting on the RNA (ADAR) protein family [5,6]. During the within-host evolution of the quasispecies, the proportions of various subpopulations undergo different changes, leading to iSNVs with diverse alternate allele frequencies (AAFs), some with high AAFs, and others with low AAFs [7,8,9,10]. When transmitted, iSNVs with higher AAFs are significantly more likely to be passed on to new hosts [11]. After the transmission process, the transmitted iSNVs engage in competition with prevalent mutations in the viral population [12,13,14]. Only through this competition can they establish themselves as new prevalent mutations, exhibiting a high proportion within the worldwide viral population.

Within these processes, it remains uncertain whether iSNVs with higher AAFs are more likely to prevail in the viral population. Additionally, if this is not the case, the question arises as to which types of iSNVs will become prevalent. In this study, we analyzed 556,843 SARS-CoV-2 sequencing datasets and 25,440 spike protein mutation prevalence records to identify key factors influencing the fate of iSNVs in the *S* gene.

## 2. Materials and Methods

### 2.1. Sequencing Data and Genomics Data

The project PRJEB37886, available in the European Nucleotide Archive (ENA) (https://www.ebi.ac.uk/ena, accessed on 27 February 2024), is a SARS-CoV-2 sequencing data-sharing project initiated by the COVID-19 Genomics UK (COG-UK) Consortium. As of 27 February 2024, it is the largest and most representative dataset of SARS-CoV-2 sequencing data, comprising 2,686,705 records. Subsequently, we applied the following criteria to filter the records to ensure the consistency and quality of the data:

1. Excluded samples with multiple sequencing data records.
2. Samples collected between 2019 and 2022 were considered.
3. Only data sequenced on the Illumina NovaSeq 6000 platform were included.
4. Selected records with paired library layout.
5. Ensured that records have corresponding assembled genome sequences in GISAID (https://gisaid.org/).
6. Genomic sequences containing unknown or degenerate bases were filtered out.

The sequencing data (BAM format) were retrieved from ENA, and the genomic sequences were downloaded from GISAID.

### 2.2. Mutations Calling

For each sequencing data, the mutations and their alternate allele frequencies (AAFs) were determined according to the following pipeline:

1. Extracting reads mapping to the *S* gene (samtools view) [15].
2. Trimming primers (ivar trim) [16].
3. Calculating the depth at each position (samtools depth) [15].
4. Calling mutations (Freebayes -p 1 -4 -V -F 0.02 -C 5 --min-coverage 10) [17].
5. Calculate the AAF for each mutation based on the depth information in the VCF format files.

As for the genomic sequence, each sequence was firstly aligned to the reference SARS-CoV-2 *S* gene (NCBI, NC_045512.2:21563-25384) by MAFFT [18]. Subsequently, the mutations of the *S* gene of each genomic sequence were detected using an in-house script.

### 2.3. Filtration of the Mutations of Sequencing Data

Sequencing data with a depth no lower than 100× at over 95% positions were included. Subsequently, the mutations of the sequencing data were further filtered based on the following criteria:

1. Each mutational position must have a depth greater than 100×.
2. There should be a minimum of 5 reads supporting the mutation.
3. The AAF of the mutation must be greater than 3%.
4. Exclusion of highly shared sites (Table S1).

Following filtration, the identified mutations of each sample were further classified into two types: single nucleotide variants (SNVs) and intra-host single nucleotide variants (iSNVs). For a sample, SNVs were defined as mutations in the *S* gene of the corresponding genomic sequence or mutations with AAFs greater than 50%. The remaining mutations of the sample were defined as iSNVs. Subsequently, samples with more than 30 iSNVs were excluded, resulting in the final dataset for this study, which consisted of 556,843 samples. This study exclusively focused on the iSNVs in the *S* gene of the samples. iSNVs were annotated using ANNOVAR [19].

### 2.4. Within-Host Selection Analysis

Within-host nucleotide diversity is estimated separately for non-synonymous ($\pi_{N}$) and synonymous ($\pi_{S}$) iSNVs at each amino acid position of the S protein, with the exclusion of the first amino acid position. In general, $\pi_{N}-\pi_{S}<0$ indicates negative selection; $\pi_{N}-\pi_{S}=0$ indicates neutral selection; and $\pi_{N}-\pi_{S}>0$ indicates positive selection.

The method used to calculate $\pi_{N}$ and $\pi_{S}$ was referred to in Ref. [20]. Briefly, we first counted the number of synonymous and non-synonymous changes (T) for each codon in the reference *S* gene. Subsequently, $\pi_{N}$ and $\pi_{S}$ at each amino acid position were calculated separately according to the following formula:

$$\frac{\sum_{i=1}^{3}\frac{A_{i}*R_{i}}{(D_{i}^{2}-D_{i})/2}}{T}\;$$

where $A_{i}$ is the number of reads supporting the iSNV at the i-th position of the codon; $R_{i}$ is the number of reference reads at the position; $D_{i}$ is the total number of reads at the position; and T is the total number of potential synonymous or non-synonymous substitutions at the codon.

### 2.5. Prevalence Data

Prevalence data for every possible mutation at each amino acid position of the S protein were obtained from outbreak.info on 10 October 2023 [21].

### 2.6. Important Epitope Regions of the S Protein

Serological analyses revealed that approximately 90% of the plasma or serum-neutralizing antibody activity targets the spike receptor-binding domain (RBD) [22]. Additionally, there are several supersites in the N-terminal domain (NTD) playing a crucial role in antigenicity [23]. For the downstream analysis, we gathered the significant epitopes of the S protein [23,24], and the sites are listed in Table S2.

## 3. Results

### 3.1. Within-Host Diversity of the SARS-CoV-2 S Gene

Given that the process of mutation creates a genetic variation that fuels evolution, we first aimed to investigate the within-host diversity of the SARS-CoV-2 *S* gene. To ensure a high-confident dataset of iSNVs, we applied stringent filtration criteria to both samples and iSNVs (see Section 2). The ultimate dataset consisted of 556,843 samples and a total of 500,441 iSNVs in the *S* gene.

**Figure 1:**
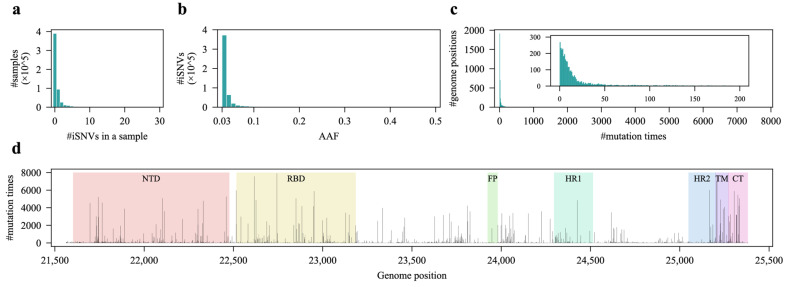
Within-host diversity of the SARS-CoV-2 *S* gene. (**a**) Distribution of the number of iSNVs detected per sample. (**b**) Distribution of alternate allele frequencies (AAFs) for all iSNVs. (**c**) The outer panel presents the distribution of mutation occurrences across each genome position, while the inner panel focuses on positions with fewer than 200 mutations, sharing the same axis labels. (**d**) Number of mutations at various positions along the S gene, with key S protein domains highlighted. NTD, amino-terminal domain; RBD, receptor-binding domain; FP, fusion peptide; HR1, heptad repeat 1; HR2, heptad repeat 2; TM, transmembrane anchor; CT, cytoplasmic tail.

Overall, most of the samples did not exhibit any iSNVs in the *S* gene. Only approximately 30% (166,843/556,843) of the samples carried at least 1 iSNV in the *S* gene, mostly fewer than 5 iSNVs, with an average of 0.90 iSNVs per sample (IQR: 0.0–1.0) (Figure 1a), indicating a low within-host diversity of the SARS-CoV-2 *S* gene. However, given the extensive infected human population, even low within-host diversity can still contribute to a substantial mutation pool for further evolution or adaptation to humans. Regarding their AAFs, the majority of the iSNVs exhibited low values (less than 0.1), with a predominant distribution between 0.03 and 0.04 (Figure 1b). This suggests that most iSNVs did not confer a higher within-host fitness advantage to the viral subpopulation compared to their corresponding raw infected viral population.

We next explored the concurrence of iSNVs, which refers to the mutation times at specific positions. The data revealed that 7% (269/3822, including the 81 masked sites (Table S1)) of the positions of the *S* gene did not undergo any mutations, while the majority of the positions (3059/3822, 80%) mutated fewer than 100 but at least 1 time in the dataset (Figure 1c). As for the distribution of the iSNVs, they were unevenly distributed across the *S* gene and displayed some mutational hotspots, particularly in some regions of the NTD, RBD, TM, and CT domains, implying a role of biased RNA-editing or selection across the *S* gene (Figure 1d).

### 3.2. The Mutational Patterns of iSNVs and Population-Level Mutations in the S Gene Exhibit a Moderate Degree of Similarity

**Figure 2:**
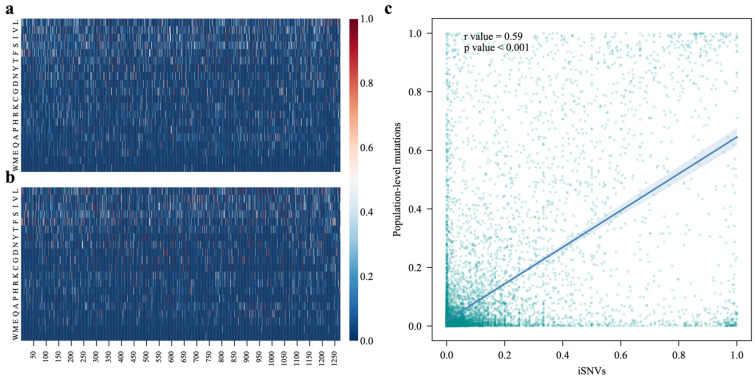
Mutational heatmap of iSNVs and population-level mutations. (**a**) Mutational heatmap of iSNVs, where each column represents an amino acid position. We counted the occurrences of the 20 possible amino acid mutations and calculated their respective proportions. (**b**) Mutational heatmap of population-level mutations, calculated similarly to the iSNV heatmap but using population-level mutation data. (**c**) Correlation of mutational proportions between iSNVs and population-level mutations for different amino acids at each amino acid position.

To investigate the similarity between iSNVs and population-level mutations in the *S* gene, we compared their mutational patterns at each amino acid position. We computed the mutational proportion to different amino acids at each amino acid position of the S protein and illustrated the mutational patterns at both within-host and population levels (Figure 2a,b). The iSNVs and population-level mutations exhibited similar mutational patterns, with a significant correlation almost approaching 0.6 (*p* < 0.001) (Figure 2a–c). Considering that mutations in the viral population originally came from iSNVs, it was reasonable to identify key factors that may contribute to the prevalence of iSNVs.

We also observed interesting mutational patterns at both within-host and population levels (Figure 2a,b). Firstly, iSNVs exhibited a broader mutational space, tending to undergo as many diverse amino acid mutations as possible at each position (Figure 2a). However, the population-level mutations showed a much narrower mutational space (Figure 2b). This could be a result of the narrow transmission bottleneck of SARS-CoV-2, as those iSNVs that do not confer a significant within-host fitness advantage are hard to transmit into the viral population [11]. Furthermore, the mutational direction also showed some inclination. For most amino acid positions, mutations predominantly occurred towards L, V, I, S, F, and T, while they less frequently mutated to Q, E, M, and W (Figure 2a,b).

### 3.3. Selection Status at Each Amino Acid Position of the S Protein

**Figure 3:**
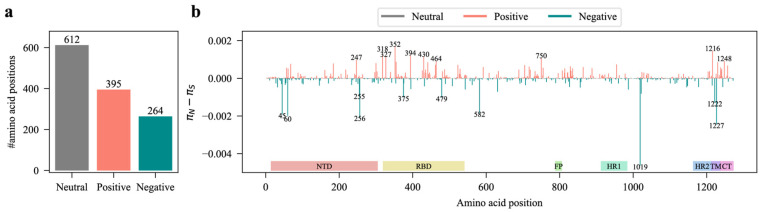
Selection status at each amino acid position of the S protein. (**a**) The number of amino acid positions under different selection statuses. (**b**) The selection status at each amino acid position across the S protein.

It is widely accepted that mutations under positive selection are more advantageous. Therefore, we characterized the selection status at each amino acid position (excluding the first amino acid of the S protein) of the SARS-CoV-2 S protein. The S protein, consisting of 1273 amino acids, had 612 amino acid positions under neutral selection, 395 under positive selection, and 264 under negative selection (Figure 3a). The selection status of the 973rd amino acid position was unrecognized due to a lack of iSNV data at this position. Consistent with the distribution of iSNVs, positions under neutral, positive, and negative selection were also spread throughout the entire S protein, exhibiting certain hotspots (Figure 3b). The hotspots for positive selection were mainly located in the NTD, RBD, TM, and CT domains (Figure 3b). We identified the top 10 amino acid positions exhibiting the strongest positive/negative selection based on the value of $\pi_{N}-\pi_{S}$ (Figure 3b). Of the 10 amino acid positions under positive selection, 6 (318, 327, 352, 394, 430, and 464) were in the RBD. However, only 2 out of the 10 positions under negative selection were in the RBD (Figure 3b). These observations suggest that SARS-CoV-2 continuously seeks to accumulate advantageous mutations in the RBD of the S protein due to the adaptive evolution to humans.

### 3.4. Association Analysis Between Factors and Prevalence of Non-Synonymous iSNVs

**Figure 4:**
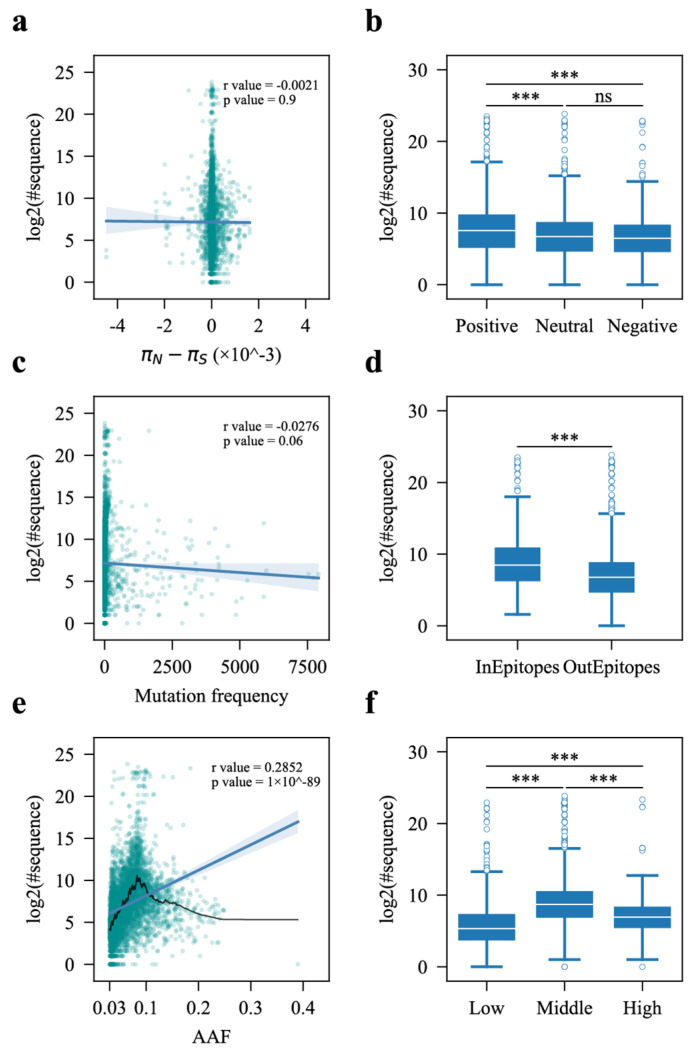
Association analysis between factors and the prevalence of non-synonymous iSNV. Association analysis between the $\pi_{N}-\pi_{S}$ values (**a**), selection statuses (**b**), mutation frequency (**c**), location (**d**), AAFs (**e**), and AAF groups (**f**) of iSNVs and their prevalence. “***” indicates a *p*-value less than 0.001, signifying statistical significance, “ns” indicates no statistical significance.

Given the moderate degree of similarity in mutational patterns between iSNVs and population-level mutations, we aimed to identify factors that contribute most to the prevalence of non-synonymous iSNVs in the *S* gene. It is widely acknowledged that mutations subject to positive selection are more likely to confer a fitness advantage. Therefore, we first focused on examining the correlation between the values of $\pi_{N}-\pi_{S}$ for iSNVs and their prevalence within the viral population. Despite not discovering any significant correlation between the $\pi_{N}-\pi_{S}$ values and prevalence, we did observe a notable difference in the prevalence of iSNVs under positive selection compared to those under neutral or negative selection (Figure 4a,b). Specifically, iSNVs under positive selection exhibited a significantly higher prevalence (Figure 4b). Moreover, there was no discernible difference in prevalence between iSNVs under neutral and negative selection (Figure 4b).

A previous study suggested that recurrent iSNVs are more likely to become prevalent [25,26]. However, our data did not reveal any significant correlation between the mutation frequency of iSNVs and their prevalence (Figure 4c). Additionally, many iSNVs with the highest mutation frequencies exhibited relatively low or moderate prevalence (Figure 4c). Therefore, recurrent iSNVs suggest that the corresponding positions are mutational hotspots [10], but this does not mean that they will exhibit a greater likelihood of prevalence.

A notable characteristic of VOC strains is their accumulation of mutations in the S protein that enhance immune escape capabilities [2,27,28,29]. Consequently, we hypothesized that iSNVs with the potential to alter the antigenicity of the S protein, allowing the virus to evade antibodies elicited by prior infections and/or vaccinations, are more likely to become prevalent. We collected known important epitope regions of the S protein, a total of 116 sites, and categorized iSNVs into two classes: iSNVs located within these regions (InEpitopes) and iSNVs outside of these regions (OutEpitopes) (Table S2) [23,24]. As anticipated, our observations revealed that iSNVs within important epitope regions exhibited a significantly higher prevalence compared to those outside of these regions (Figure 4d). This finding suggests that iSNVs within important epitope regions are much more likely to enhance immune escape abilities and confer a growth advantage to the variants.

The alternate allele frequency (AAF) of an iSNV represents the proportion of the viral subpopulation carrying the iSNV and can be considered as a metric of within-host fitness of the iSNV relative to their ancestral variants within the quasispecies. It has been demonstrated that iSNVs with higher AAFs are more likely to transmit [11]. However, the following question arises: does a higher AAF also imply a greater likelihood of prevalence? To explore this, we investigated the relationship between AAF and prevalence. Our analysis revealed a significant positive correlation between the prevalence of iSNVs and their AAFs (the blue line, r = 0.29, *p* < 0.001, Figure 4e). We further smoothed the curve as AAFs increased (Figure 4e, the black line) and observed that iSNVs with moderate AAFs were likely to exhibit higher prevalence. Consequently, we classified iSNVs into three groups based on their AAFs: iSNVs with AAFs lower than 0.06 (low), iSNVs with AAFs between 0.06 and 0.12 (middle), and iSNVs with AAFs greater than 0.12 (high). The prevalence of iSNVs in the middle group was significantly higher than those in the low and high groups (Figure 4f). Additionally, the prevalence of iSNVs in the high group was notably higher than that in the low group (Figure 4f). These results suggest that the within-host fitness of iSNVs contributes to their prevalence, and iSNVs with moderate within-host fitness, rather than high within-host fitness, are more likely to be prevalent.

In summary, three factors, selection status, antigenicity shift, and within-host fitness, are potential contributors to the prevalence of non-synonymous iSNVs in the *S* gene.

### 3.5. Key Factors Influencing the Prevalence of Non-Synonymous iSNVs in the S Gene

Above, we found that selection status, antigenicity shift, and within-host fitness may contribute to the prevalence of iSNVs. The analysis of variance (ANOVA) revealed that all three factors had a statistically significant influence on prevalence (Table S3). The multiple linear regression analysis demonstrated that various factors contribute differently to the prevalence of iSNVs. Among the three factors, within-host fitness exhibited the greatest contribution, followed by antigenicity shift, while selection status had the least impact. Furthermore, compared to the other two factors, selection status displayed one order of magnitude lower influence on the prevalence of iSNVs. Consequently, within-host fitness and antigenicity shift are the two most important factors.

**Figure 5:**
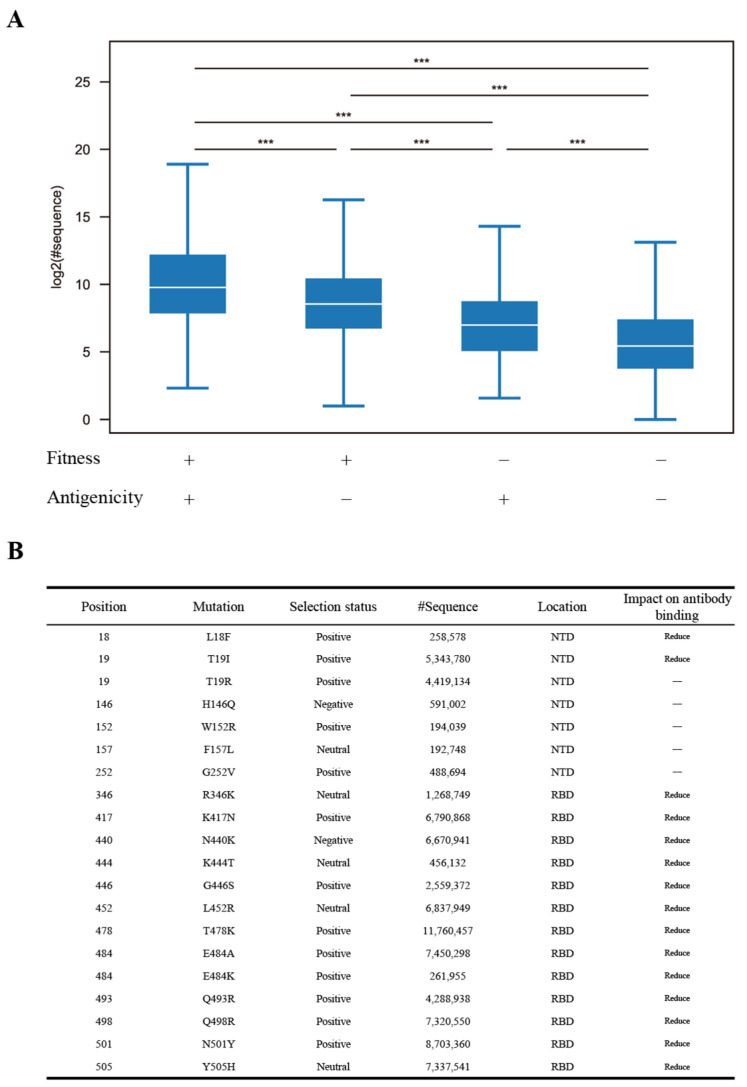
Key factors influencing the prevalence of iSNVs. (**A**) Differences in prevalence between different iSNV groups. “+” represents iSNVs with moderate within-host fitness or located in significant epitope regions; “−” represents iSNVs without moderate within-host fitness or located out of significant epitope regions. (**B**) The top 20 iSNVs exhibiting the highest prevalence and their impact on antibody binding. “***” indicates a *p*-value less than 0.001 signifying statistical significance.

Considering that iSNVs with moderate within-host fitness exhibited the greatest prevalence and iSNVs within important epitope regions showed higher prevalence than those outside of these regions, we classified non-synonymous iSNVs in the *S* gene into four groups based on whether they have moderate within-host fitness and whether they are in important epitope regions. Our observations revealed that iSNVs with moderate within-host fitness and located in important epitope regions exhibited much higher prevalence than iSNVs in any other groups (Figure 5A). In contrast, iSNVs that lacked both moderate within-host fitness and presence in important epitope regions displayed the lowest prevalence (Figure 5A). This result indicates that iSNVs with the features of moderate within-host fitness and located in important epitope regions have the most potential to become prevalent.

We then conducted a detailed examination of iSNVs exhibiting moderate within-host fitness and located in important epitope regions of the S protein, including 254 distinct non-synonymous mutations occurring at 102 amino acid positions. Most of these positions exhibited one to four unique amino acid mutations, while only three positions (the 261st, 483rd, and 484th amino acid positions) showed more than four amino acid mutations (Table S4). Among the 254 iSNVs, 149 were subjected to positive selection, 72 to neutral selection, and only 33 to negative selection (Table S4). F157I had the lowest prevalence, appearing in only 5 genome sequences, whereas T478K had the highest prevalence, appearing in 11,760,457 genome sequences. Additionally, 49 iSNVs each appeared in at least 10,000 genome sequences (Table S4). We listed the top 20 iSNVs with the highest prevalence (Figure 5B). Among these 20 iSNVs, 7 were in the NTD, while the remaining 13 were in the RBD (Figure 5B). NTD iSNVs L18F and T19I were shown to reduce neutralization by NTD-targeted antibodies (Figure 5B) [30,31], while all the RBD iSNVs were associated with decreased neutralization by antibodies, thereby enhancing the immune escape ability of SARS-CoV-2 (Figure 5B) [31,32,33,34,35,36,37,38].

## 4. Discussion

In the present study, we conducted a systematic analysis of the within-host diversity of the SARS-CoV-2 *S* gene and generated a single amino acid resolution selection status map of the S protein. Additionally, we investigated several factors that may influence the prevalence of non-synonymous iSNVs in the *S* gene. Our findings indicate that within-host fitness and antigenicity shift are two key factors that largely influence whether an iSNV will become prevalent or not.

To achieve widespread prevalence, new variants must effectively evade infection-induced and vaccine-elicited neutralizing antibodies, especially in the era of the circulation of the Omicron variant and its descendant lineages [33,39,40,41,42]. We previously revealed that a considerable proportion of non-synonymous iSNVs in the *S* gene can alter the antigenic features of the S protein [43]. In the present study, we observed that iSNVs located in important epitope regions of the S protein are much more likely to become prevalent (Figure 4d). These iSNVs have the potential to alter the antigenicity of the S protein, thus enhancing the capability of immune escape, which provides the variants with a growth advantage within the viral population.

We also observed that most amino acid positions of the S protein were under neutral or positive selection in terms of iSNVs (Figure 3), which is consistent with the findings that positive selection occurs on the iSNVs of the *S* gene [44]. These observations together indicate a within-host fitness selection acting on iSNVs. The AAFs of iSNVs show a wide distribution range, indicating a significant variance in within-host fitness among iSNVs (Figure 1b and Figure 4e). Moreover, the presence of a considerable number of iSNVs with relatively low AAFs suggests that many iSNVs confer a minimal to no within-host fitness advantage (Figure 1b and Figure 4e). Almost all these iSNVs will be eliminated due to the narrow transmission bottleneck of SARS-CoV-2 [11].

Studies on the transmission of iSNVs have demonstrated that those with higher AAFs are more likely to transmit [11]. Intuitively, one might expect these types of iSNVs, as well as recurrent iSNVs, to be more likely to become prevalent because of a higher likelihood of transmission. However, our data do not support this intuition (Figure 4c,e,f). Instead, iSNVs with middle AAFs showed a greater likelihood of becoming prevalent in the viral population compared to iSNVs with a high AAF (Figure 4f). This discrepancy indicates a different selection process acting on the within-host level and interhost-level evolution, and the selective pressure on the two evolution stages seems to be antagonistic to some extent [44,45]. This can explain why iSNVs with high AAFs are less likely to become prevalent.

Taken together, we can conclude that for an iSNV to become prevalent, it should possess two key features. First, it should confer some within-host fitness advantage, ensuring that the AAF of the iSNV increases to a transmissible frequency. Second, it should be capable of altering antigenicity to enhance the immune escape ability of the variant, thus conferring a growth advantage when competing with other circulating variants in the viral population. Due to the antagonistic nature of selective pressure between the two evolution stages, iSNVs exhibiting a growth advantage in the viral population do not necessarily confer a high within-host fitness. This possibly explains why highly prevalent iSNVs were clustered in the middle AAF group.

In conclusion, iSNVs with a high AAF do not guarantee prevalence in the viral population, and iSNVs with a middle AAF are more prevalent. Within-host fitness and antigenicity shift are key factors influencing the prevalence of iSNVs.

## Data Availability

All sequencing data were retrieved from a public database (ENA, project PRJEB37886). All other data are available in the main text and Supplementary Materials.

## Supplementary Materials

The following supporting information can be downloaded at: https://www.mdpi.com/article/10.3390/v17030362/s1, Table S1. The list of masked sites; Table S2. The list of significant epitope regions of the SARS-CoV-2 S protein; Table S3. Variance analysis of different factors; Table S4. The list of iSNVs with moderate within-host fitness and capable of changing antigenicity.

## References

[1] Obermeyer F., Jankowiak M., Barkas N., Schaffner S.F., Pyle J.D., Yurkovetskiy L., Bosso M., Park D.J., Babadi M., MacInnis B.L. (2022). Analysis of 6.4 million SARS-CoV-2 genomes identifies mutations associated with fitness. Science.

[2] Harvey W.T., Carabelli A.M., Jackson B., Gupta R.K., Thomson E.C., Harrison E.M., Ludden C., Reeve R., Rambaut A., Peacock S.J. (2021). SARS-CoV-2 variants, spike mutations and immune escape. Nat. Rev. Microbiol..

[3] Tao K., Tzou P.L., Nouhin J., Gupta R.K., de Oliveira T., Kosakovsky Pond S.L., Fera D., Shafer R.W. (2021). The biological and clinical significance of emerging SARS-CoV-2 variants. Nat. Rev. Genet..

[4] Sun F., Wang X., Tan S., Dan Y., Lu Y., Zhang J., Xu J., Tan Z., Xiang X., Zhou Y. (2021). SARS-CoV-2 quasispecies provides an advantage mutation pool for the epidemic variants. Microbiol. Spectr..

[5] Mourier T., Sadykov M., Carr M.J., Gonzalez G., Hall W.W., Pain A. (2021). Host-directed editing of the SARS-CoV-2 genome. Biochem. Biophys. Res. Commun..

[6] Giorgio1 S.D., Martignano1 F., Torcia M.G., Mattiuz1 G., Conticello1 S.G. (2020). Evidence for host-dependent RNA editing in the transcriptome of SARS-CoV-2. Sci. Adv..

[7] Wang Y., Wang D., Zhang L., Sun W., Zhang Z., Chen W., Zhu A., Huang Y., Xiao F., Yao J. (2021). Intra-host variation and evolutionary dynamics of SARS-CoV-2 populations in COVID-19 patients. Genome Med..

[8] Farjo M., Koelle K., Martin M.A., Gibson L.L., Walden K.K.O., Rendon G., Fields C.J., Alnaji F.G., Gallagher N., Luo C.H. (2024). Within-host evolutionary dynamics and tissue compartmentalization during acute SARS-CoV-2 infection. J. Virol..

[9] Gonzalez-Reiche A.S., Alshammary H., Schaefer S., Patel G., Polanco J., Carreño J.M., Amoako A.A., Rooker A., Cognigni C., Floda D. (2023). Sequential intrahost evolution and onward transmission of SARS-CoV-2 variants. Nat. Commun..

[10] Tonkin-Hill G., Martincorena I., Amato R., Lawson A.R., Gerstung M., Johnston I., Jackson D.K., Park N., Lensing S.V., Quail M.A. (2021). Patterns of within-host genetic diversity in SARS-COV-2. eLife.

[11] Lythgoe K.A., Hall M., Ferretti L., de Cesare M., MacIntyre-Cockett G., Trebes A., Andersson M., Otecko N., Wise E.L., Moore N. (2021). SARS-CoV-2 within-host diversity and transmission. Science.

[12] Markov P.V., Ghafari M., Beer M., Lythgoe K., Simmonds P., Stilianakis N.I., Katzourakis A. (2023). The evolution of SARS-CoV-2. Nat. Rev. Microbiol..

[13] Eales O., de Oliveira Martins L., Page A.J., Wang H., Bodinier B., Tang D., Haw D., Jonnerby J., Atchison C., Ashby D. (2022). Dynamics of competing SARS-CoV-2 variants during the Omicron epidemic in England. Nat. Commun..

[14] Boyle L., Hletko S., Huang J., Lee J., Pallod G., Tung H.-R., Durrett R. (2022). Selective sweeps in SARS-CoV-2 variant competition. Proc. Natl. Acad. Sci. USA.

[15] Danecek P., Bonfield J.K., Liddle J., Marshall J., Ohan V., Pollard M.O., Whitwham A., Keane T., McCarthy S.A., Davies R.M. (2021). Twelve years of SAMtools and BCFtools. Gigascience.

[16] Grubaugh N.D., Gangavarapu K., Quick J., Matteson N.L., De Jesus J.G., Main B.J., Tan A.L., Paul L.M., Brackney D.E., Grewal S. (2019). An amplicon-based sequencing framework for accurately measuring intrahost virus diversity using PrimalSeq and iVar. Genome Biol..

[17] Garrison E., Marth G. (2012). Haplotype-based variant detection from short-read sequencing. arXiv.

[18] Katoh K., Misawa K., Kuma K.I., Miyata T. (2002). MAFFT: A novel method for rapid multiple sequence alignment based on fast Fourier transform. Nucleic Acids Res..

[19] Wang K., Li M., Hakonarson H. (2010). ANNOVAR: Functional annotation of genetic variants from high-throughput sequencing data. Nucleic Acids Res..

[20] Nelson C.W., Hughes A.L. (2015). Within-host nucleotide diversity of virus populations: Insights from next-generation sequencing. Infect. Genet. Evol..

[21] Gangavarapu K., Latif A.A., Mullen J.L., Alkuzweny M., Hufbauer E., Tsueng G., Haag E., Zeller M., Aceves C.M., Zaiets K. (2023). Outbreak.info genomic reports: Scalable and dynamic surveillance of SARS-CoV-2 variants and mutations. Nat. Methods.

[22] Piccoli L., Park Y.-J., Tortorici M.A., Czudnochowski N., Walls A.C., Beltramello M., Silacci-Fregni C., Pinto D., Rosen L.E., Bowen J.E. (2020). Mapping neutralizing and immunodominant sites on the SARS-CoV-2 spike receptor-binding domain by structure-guided high-resolution serology. Cell.

[23] McCallum M., Marco A.D., Lempp F.A., Tortorici M.A., Pinto D., Walls A.C., Beltramello M., Chen A., Liu Z., Zatta F. (2021). N-terminal domain antigenic mapping reveals a site of vulnerability for SARS-CoV-2. Cell.

[24] Yisimayi A., Song W., Wang J., Jian F., Yu Y., Chen X., Xu Y., Yang S., Niu X., Xiao T. (2024). Repeated Omicron exposures override ancestral SARS-CoV-2 immune imprinting. Nature.

[25] Gu H., Quadeer A.A., Krishnan P., Ng D.Y.M., Chang L.D.J., Liu G.Y.Z., Cheng S.M.S., Lam T.T.Y., Peiris M., McKay M.R. (2023). Within-host genetic diversity of SARS-CoV-2 lineages in unvaccinated and vaccinated individuals. Nat. Commun..

[26] Wilkinson S.A.J., Richter A., Casey A., Osman H., Mirza J.D., Stockton J., Quick J., Ratcliffe L., Sparks N., Cumley N. (2022). Recurrent SARS-CoV-2 mutations in immunodeficient patients. Virus Evol..

[27] Hu J., Peng P., Cao X., Wu K., Chen J., Wang K., Tang N., Huang A. (2022). Increased immune escape of the new SARS-CoV-2 variant of concern Omicron. Cell. Mol. Immunol..

[28] Carabelli A.M., Peacock T.P., Thorne L.G., Harvey W.T., Hughes J., de Silva T.I., Peacock S.J., Barclay W.S., de Silva T.I., Towers G.J. (2023). SARS-CoV-2 variant biology: Immune escape, transmission and fitness. Nat. Rev. Microbiol..

[29] Cai Y., Zhang J., Xiao T., Lavine C.L., Rawson S., Peng H., Zhu H., Anand K., Tong P., Gautam A. (2021). Structural basis for enhanced infectivity and immune evasion of SARS-CoV-2 variants. Science.

[30] Dejnirattisai W., Zhou D., Supasa P., Liu C., Mentzer A.J., Ginn H.M., Zhao Y., Duyvesteyn H.M.E., Tuekprakhon A., Nutalai R. (2021). Antibody evasion by the P.1 strain of SARS-CoV-2. Cell.

[31] Ai J., Wang X., He X., Zhao X., Zhang Y., Jiang Y., Li M., Cui Y., Chen Y., Qiao R. (2022). Antibody evasion of SARS-CoV-2 Omicron BA.1, BA.1.1, BA.2, and BA.3 sub-lineages. Cell Host Microbe.

[32] Chen J., Gao K., Wang R., Wei G.W. (2021). Revealing the threat of emerging SARS-CoV-2 mutations to antibody therapies. J. Mol. Biol..

[33] Cui Z., Liu P., Wang N., Wang L., Fan K., Zhu Q., Wang K., Chen R., Feng R., Jia Z. (2022). Structural and functional characterizations of infectivity and immune evasion of SARS-CoV-2 Omicron. Cell.

[34] Qu P., Evans J.P., Faraone J.N., Zheng Y.-M., Carlin C., Anghelina M., Stevens P., Fernandez S., Jones D., Lozanski G. (2023). Enhanced neutralization resistance of SARS-CoV-2 Omicron subvariants BQ.1, BQ.1.1, BA.4.6, BF.7, and BA.2.75.2. Cell Host Microbe.

[35] Dadonaite B., Crawford K.H.D., Radford C.E., Farrell A.G., Yu T.C., Hannon W.W., Zhou P., Andrabi R., Burton D.R., Liu L. (2023). A pseudovirus system enables deep mutational scanning of the full SARS-CoV-2 spike. Cell.

[36] Wang Q., Iketani S., Li Z., Liu L., Guo Y., Huang Y., Bowen A.D., Liu M., Wang M., Yu J. (2023). Alarming antibody evasion properties of rising SARS-CoV-2 BQ and XBB subvariants. Cell.

[37] Deng X., Garcia-Knight M.A., Khalid M.M., Servellita V., Wang C., Morris M.K., Sotomayor-González A., Glasner D.R., Reyes K.R., Gliwa A.S. (2021). Transmission, infectivity, and neutralization of a spike L452R SARS-CoV-2 variant. Cell.

[38] Jangra S., Ye C., Rathnasinghe R., Stadlbauer D., Alshammary H., Amoako A.A., Awawda M.H., Beach K.F., Bermúdez-González M.C., Chernet R.L. (2021). SARS-CoV-2 spike E484K mutation reduces antibody neutralisation. Lancet Microbe.

[39] McCallum M., Czudnochowski N., Rosen L.E., Zepeda S.K., Bowen J.E., Walls A.C., Hauser K., Joshi A., Stewart C., Dillen J.R. (2022). Structural basis of SARS-CoV-2 Omicron immune evasion and receptor engagement. Science.

[40] Uraki R., Ito M., Furusawa Y., Yamayoshi S., Iwatsuki-Horimoto K., Adachi E., Saito M., Koga M., Tsutsumi T., Yamamoto S. (2023). Humoral immune evasion of the Omicron subvariants BQ.1.1 and XBB. Lancet Infect. Dis..

[41] Zhang X., Wu S., Wu B., Yang Q., Chen A., Li Y., Zhang Y., Pan T., Zhang H., He X. (2021). SARS-CoV-2 Omicron strain exhibits potent capabilities for immune evasion and viral entrance. Signal Transduct. Target. Ther..

[42] Xia S., Wang L., Zhu Y., Lu L., Jiang S. (2022). Origin, virological features, immune evasion and intervention of SARS-CoV-2 Omicron sublineages. Signal Transduct. Target. Ther..

[43] Xi B., Zeng X., Chen Z., Zeng J., Huang L., Du H. (2023). SARS-CoV-2 within-host diversity of human hosts and its implications for viral immune evasion. mBio.

[44] Li J., Du P., Yang L., Zhang J., Song C., Chen D., Song Y., Ding N., Hua M., Han K. (2022). Two-step fitness selection for intra-host variations in SARS-CoV-2. Cell Rep..

[45] Hou M., Shi J., Gong Z., Wen H., Lan Y., Deng X., Fan Q., Li J., Jiang M., Tang X. (2023). Intra- vs. interhost evolution of SARS-CoV-2 driven by uncorrelated selection—The evolution thwarted. Mol. Biol. Evol..

